# A distal super-enhancer activates oncogenic *ETS2* via recruiting MECOM in inflammatory bowel disease and colorectal cancer

**DOI:** 10.1038/s41419-022-05513-1

**Published:** 2023-01-06

**Authors:** Yongheng Chen, Ying Ying, Maolin Wang, Canjie Ma, Min Jia, Liang Shi, Shilan Wang, Xiangyi Zheng, Wei Chen, Xing-sheng Shu

**Affiliations:** 1 Department of Physiology, School of Basic Medical Sciences, Health Science Center, Shenzhen University, Shenzhen, 518060 China; 2 School of Pharmaceutical Sciences, Health Science Center, Shenzhen University, Shenzhen, 518060 China; 3 Marshall Laboratory of Biomedical Engineering, Health Science Center, Shenzhen University, Shenzhen, 518060 China; 4 Clinical Research Center, The First Affiliated Hospital of Shantou University Medical College, Shantou, 515000 China

**Keywords:** Cancer epigenetics, Colorectal cancer

## Abstract

Abnormal activities of distal cis-regulatory elements (CREs) contribute to the initiation and progression of cancer. Gain of super-enhancer (SE), a highly active distal CRE, is essential for the activation of key oncogenes in various cancers. However, the mechanism of action for most tumor-specific SEs still largely remains elusive. Here, we report that a candidate oncogene *ETS2* was activated by a distal SE in inflammatory bowel disease (IBD) and colorectal cancer (CRC). The SE physically interacted with the *ETS2* promoter and was required for the transcription activation of *ETS2*. Strikingly, the *ETS2*-SE activity was dramatically upregulated in both IBD and CRC tissues when compared to normal colon controls and was strongly correlated with the level of *ETS2* expression. The tumor-specific activation of *ETS2*-SE was further validated by increased enhancer RNA transcription from this region in CRC. Intriguingly, a known IBD-risk SNP resides in the *ETS2*-SE and the genetic variant modulated the level of *ETS2* expression through affecting the binding of an oncogenic transcription factor MECOM. Silencing of *MECOM* induced significant downregulation of *ETS2* in CRC cells, and the level of *MECOM* and *ETS2* correlated well with each other in CRC and IBD samples. Functionally, MECOM and ETS2 were both required for maintaining the colony-formation and sphere-formation capacities of CRC cells and MECOM was crucial for promoting migration. Taken together, we uncovered a novel disease-specific SE that distantly drives oncogenic *ETS2* expression in IBD and CRC and delineated a mechanistic link between non-coding genetic variation and epigenetic regulation of gene transcription.

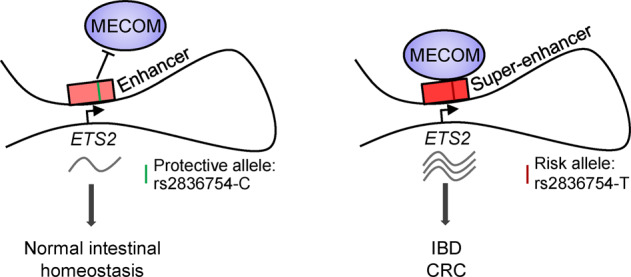

## Introduction

In eukaryote, transcriptional signature in proper temporospatial pattern is mainly determined by regulatory factors that act coordinatively in cis or trans mode [1, 2]. As one of the classic distal cis-regulatory elements, enhancer fine-tunes the level of gene transcriptional output in response to biological stimuli by controlling the dynamics for loading of transcription factors and shaping local chromatin environment around proximal promoter region [3]. Thus, in contrast to conservative promoters, enhancer activities are highly versatile and context-dependent, providing the necessary complexity for gene regulation under various biological settings. Super-enhancer (SE), defined as a subgroup of enhancers with exceptionally high activity across a larger genomic span than typical enhancers (TEs), was found to be essential for controlling the expression of key cell identify genes and cancer genes [4] probably through condensing transcription apparatus around local chromatin via liquid-liquid phase separation [5]. Intriguingly, disease-associated SNPs are more enriched in SEs than in TEs, and the enrichment is only confined to disease-relevant cell or tissue types [4]. However, how the genetic variation functionally interacts with SE regulation during disease development and progression still largely remains elusive.

Transcription regulation by distal regulatory elements is mainly achieved by the precise spatial organization of the genome in human cells. Recent advances in the 3D-genome techniques greatly extended our understanding about the long-range regulation of enhancer to target promoter(s), providing ample resource for annotating the regulatory elements in the non-coding genome [6–8]. In the meantime, although previous functional genomics studies pinpointed genetic polymorphisms associated with variation in gene expression levels (expression quantitative trait loci or eQTLs) [9], the underlying mechanistic link between the gene-eQTL pair is unclear in most cases as the variant site is usually located in distal non-coding region from its associated gene. Therefore, annotation of eQTL with the regulatory potential in cis and spatial genome organization information will benefit the dissection of genotype to expression phenotype association.

Altered epigenomic landscapes has been recognized as a fundamental driver for tumorigenesis and could be targeted for cancer therapeutics [10]. The activity of enhancers, commonly marked by the acetylation of histone H3 at lysine 27 (H3K27ac), is frequently misregulated in cancers [11]. In particular, aberrant SE activity has been found to be important for the activation of certain oncogenes [4, 12]. We and others previously showed that distorted enhancer signature, especially the gain of tumor-specific SE activity, is a key feature of colorectal cancer (CRC) [13–17], a malignancy with poor prognostic outcome prevalent worldwide [18]. Similarly, enhancer profiling in inflammatory bowel disease (IBD), a disease with high risk for developing CRC, identified variant enhancer loci enriched with IBD-associated SNPs [19, 20]. However, our knowledge about the precise functional consequence of enhancer reprogramming and the underlying molecular mechanism in CRC and IBD is still very limited. Here, we report a novel disease-specific SE in IBD and CRC that drives oncogenic *ETS2* expression through long-range regulation and further elucidated how disease-associated genetic variation modulates the transcription regulation via the *ETS2*-SE.

## Results

### The proto-oncogene *ETS2* is overexpressed in CRC and IBD samples

**Fig. 1:**
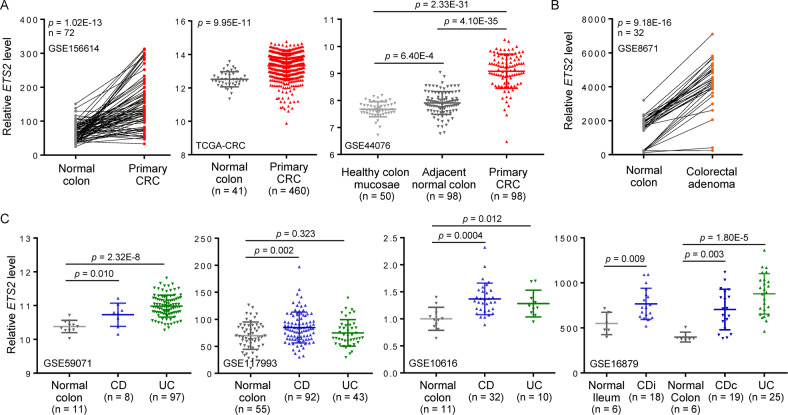
The proto-oncogene *ETS2* is overexpressed in CRC and IBD samples. **A** Relative *ETS2* mRNA expression levels in three independent primary CRC datasets with adjacent normal colon or distal healthy colon mucosae as controls. **B** Expression of *ETS2* in begin colorectal adenomas in comparison to adjacent normal colon. **C** Relative *ETS2* mRNA expression levels in four independent IBD datasets. CD Crohn’s disease, UC ulcerative colitis, CDi Crohn’s disease in ileum, CDc Crohn’s disease in colon.

In search for potential genes involved in the pathogenesis of CRC as well as the CRC-predisposed disease IBD, we identified *ETS2*, a gene coding for an evolutionarily conserved transcription factor that plays crucial roles in a plethora of physiological and pathological processes [21], as a candidate disease-associated gene. The expression of *ETS2* was significantly upregulated in primary CRC tissues when compared to adjacent normal colon or healthy colon mucosae from distal area (Fig. 1A). Moreover, *ETS2* expression was increased in colorectal adenoma (Fig. 1B), indicating that it is likely to be involved in the early development of CRC. In addition, the *ETS2* level was significantly higher in CRC patients with low mutation burden and low microsatellite instability (Supplementary Fig. 1–2), suggesting that *ETS2* might be misregulated by non-genetic pathways in CRC. Interestingly, we also observed higher *ETS2* expression in both Crohn’s disease (CD) and ulcerative colitis (UC), two major types of IBD, than in normal colon controls (Fig. 1C). These results inspired us to further study the mechanism of *ETS2* activation and its functional role in CRC and IBD.

### A distal super-enhancer activates *ETS2* transcription through long-range interaction in CRC cell lines

**Fig. 2:**
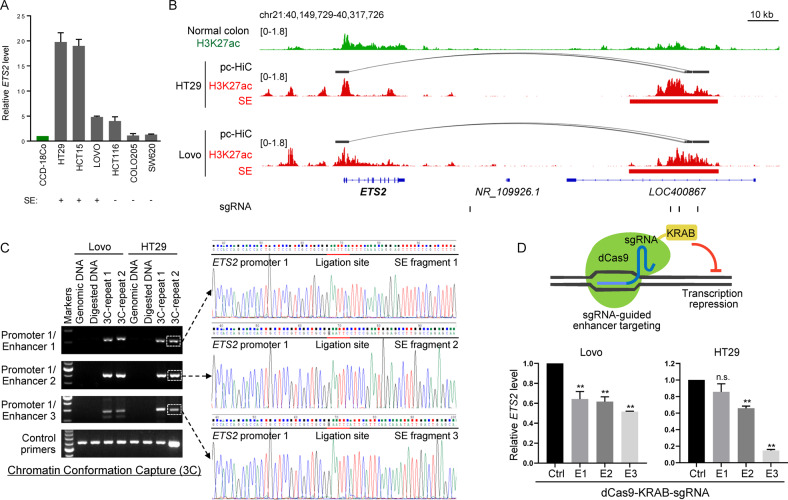
A distal super-enhancer activates *ETS2* transcription through long-range interaction in CRC cell lines. **A** Expression level of *ETS2* in CRC cell lines and a normal colonic epithelial cell line CCD-18Co as determined by RT-qPCR. SE, super-enhancer. **B** IGV tracks showing the H3K27ac ChIP-seq profiles of indicated samples across *ETS2* promoter and its potential distal SE. Bridging curves indicate the interactions between the distal SE and *ETS2* promoter detected by pc-HiC experiments in indicated cell lines. The sites targeted by guide RNAs that recruits dCas9-KRAB were indicated at the bottom. **C** Left panel: PCR results for 3C samples or control samples using primers targeting indicated regions (fragments in *ETS2* promoter or its distal SE). A pair of primers that amplifying a region nearby *ETS2* promoter that does not contain any EcoRI restriction site inside were used as positive controls. Right panel: Sanger sequencing results of indicated PCR products from 3C experiments. **D** Upper panel: diagram showing the principle of the dCas9-KRAB targeted transcription repression system. Bottom panel: expression level of *ETS2* in cells expressing sgRNAs targeting indicated enhancer fragments or a control intergenic region together with the dCas9-KRAB fusion protein. **p < 0.01; n.s. non-significant.

By integrating the epigenome (histone modification profiles) [22, 23] and 3D genome (promoter capture Hi-C) [7] data in CRC cell lines, we identified a distal SE located around 120 kb 3’ downstream from the transcription start site of *ETS2*, which was likely to interact with the *ETS2* promoter and correlated with higher *ETS2* expression in CRC cell lines with the presence of the SE (Fig. 2A, B). Chromatin Conformation Capture (3C) experiments that detect the interaction between DNA fragments through in-situ enzymatic digestion and proximity-based ligation confirmed the physical association of two independent *ETS2* promoter fragments with three different fragments within the distal SE (Fig. 2C and Supplementary Fig. 3A). To investigate whether the distal SE was required for transcription activation of *ETS2*, we used a targeted transcription repression approach based on a modified CRISPR/Cas9 system, in which the repressive KRAB domain is fused with dCas9 (a derivative form of Cas9 protein with deactivated enzymatic activity and retained site-specific DNA-binding activity) and recruited to target site by sequence-specific guide RNA [24]. The results showed that inhibition of the distal SE activity led to the downregulation of *ETS2* expression in CRC cells (Fig. 2D), suggesting that *ETS2* transcription is, at least partially, dependent on the SE.

### The *ETS2*-SE is highly tumor-specific and its activity is strongly correlated with the expression level of *ETS2* in primary CRC

**Fig. 3:**
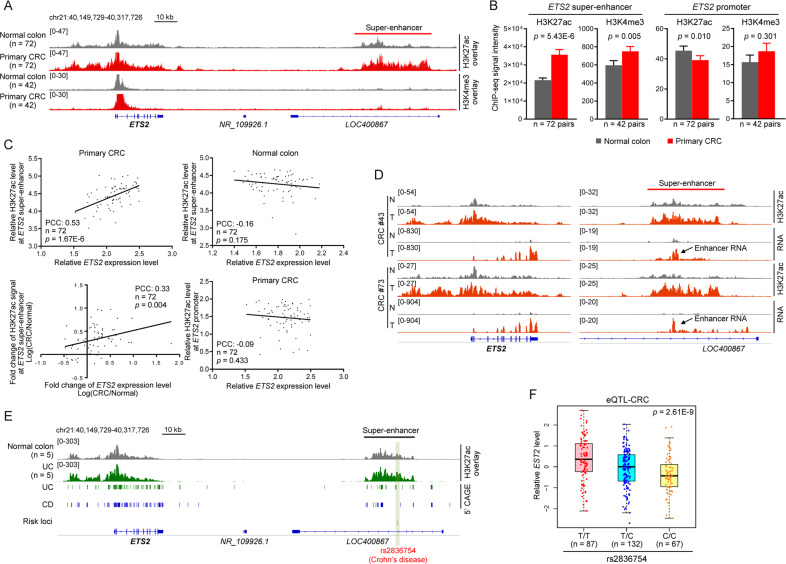
The *ETS2*-SE is highly disease-specific in both CRC and IBD and its activity is strongly correlated with the expression level of *ETS2* in CRC. **A** Overlayed IGV tracks showing the H3K27ac (*n* = 72) and H3K4me3 (*n* = 42) ChIP-seq profiles in paired normal colon (gray) and primary CRC samples (red) across the *ETS2* promoter and its distal SE. **B** Quantification of the ChIP-seq signal (RPGC in a bin size of 10 bp) from samples described in (**A**) at *ETS2* promoter or its distal SE. **C** Scatter plots showing the correlation between *ETS2* expression level and H3K27ac level at *ETS2*-SE or *ETS2* promoter in primary CRC or normal colon tissues. PCC Pearson Correlation Coefficient. **D** IGV tracks showing the H3K27ac ChIP-seq and RNA-seq profiles in normal colon tissue (N) or primary tumor (T) from two CRC patients at indicated regions. Loci of active enhancer transcription in CRC were indicated by black arrows. **E** Upper panel: IGV tracks showing the overlayed H3K27ac ChIP-seq profiles in normal colon tissue (*n* = 5, gray) or UC colon tissue (*n* = 5, dark green) at indicated regions. Bottom panel: 5’ CAGE peaks labeling active transcription events from two representative UC and CD samples. The position of a known IBD-risk SNP was indicated by green rectangle in shadow. **F** Results of eQTL analysis in primary CRCs showing differential *ETS2* expression level in samples with different risk SNP genotype.

To further explore the clinical relevance of the novel *ETS2*-SE, we obtained and reanalyzed the epigenome data of CRC patients from a large cohort study [14] with a focus on distal SEs and found that the level of H3K27ac across the *ETS2*-SE was significantly higher in primary CRC tissues than in matched normal colon tissues (*p* = 5.43E-6, *n* = 72) (Fig. 3A, B). In contrast, the level of H3K27ac around *ETS2* promoter region did not show noticeable variation between normal and tumor samples (Fig. 3B). In line with this, the level of H3K4me3, an active histone mark for promoter activity, at *ETS2*-SE remained unchanged in primary CRCs (Fig. 3B). Strikingly, the H3K27ac level at the *ETS2*-SE was strongly correlated with the level of *ETS2* expression in primary CRC but not in normal colon tissues, and the change of *ETS2*-SE activity between matched CRC and normal colon tissues largely resembled the pattern of *ETS2* expression level variation in the same samples (Fig. 3C). On the contrary, *ETS2* expression level showed no association with the H3K27ac level at *ETS2* promoter (Fig. 3C). In concert with this, we did not observe any correlation between *ETS2* and H3K4me3 levels at *ETS2*-SE or promoter (Supplementary Fig. 3B). Therefore, the dramatic increase of H3K27ac activity at *ETS2*-SE region and its strong correlation with the upregulation of *ETS2* mRNA level in primary CRC samples substantially support the hypothesis that gain of enhancer activity represents a major contributor to *ETS2* activation in CRC pathogenesis.

Upon activation, enhancers are actively transcribed by transcription factor-based recruitment of RNA polymerase II, generating enhancer RNAs (eRNAs) that facilitate target gene transcription [25]. Recent studies demonstrated that eRNA level can be accurately quantified by RNA sequencing (RNA-seq) and the pattern of eRNA expression are informative for explaining cancer phenotypes and the underlying transcription regulatory mechanisms [26, 27]. In this regard, we found that the *ETS2*-SE was actively transcribed in primary CRC tissues but not in matched normal colon tissues and the upregulation of its eRNA level was accompanied by increased transcription of *ETS2* in the same samples (Fig. 3D). Consistently, the expression level of *ETS2* was positively correlated with the eRNA level of the distal SE in CRC samples (Supplementary Fig. 3C). Together, these data suggest that gain of *ETS2*-SE is a common feature of CRC and strongly correlated with the activation of *ETS2* in clinical samples.

### An IBD risk SNP resides in the *ETS2*-SE and is an eQTL for *ETS2* in CRC

Apart from CRC samples, we also looked in the IBD samples for potential role of the *ETS2*-SE, and found that the activity of the *ETS2*-SE was indeed higher in UC organoids than in normal colon controls and the region was also actively transcribed in both UC and CD samples as detected by 5’ CAGE (Cap Analysis Gene Expression) experiments (Fig. 3E). In order to examine whether the *ETS2*-SE accounts for the functional consequence of certain non-coding variation events in IBD, we collected all known risk SNPs for IBD derived from previous GWAS (genome-wide association study) results and labelled their position within the regulatory regions of *ETS2*. As a result, a known risk SNP for CD (rs2836754; T/C) [28] was found to reside in a fragment of *ETS2*-SE (Fig. 3E) which was required for the transcription activation of *ETS2* (fragment E3 in Fig. 2D). Moreover, eQTL (expression quantitative trait loci) analysis that provides explanation for a fraction of the genetic variance of a gene expression phenotype in the PancanQTL database [9] revealed strong association between the genotype of risk SNP and *ETS2* expression level in CRC samples (Fig. 3F). Based on these findings, we speculated that the genetic polymorphism may interfere with *ETS2* transcription through altering the *ETS2*-SE activity, which encouraged us to carry the following studies.

### Oncogenic transcription factor MECOM is recruited to the *ETS2*-SE for *ETS2* transcription activation

**Fig. 4:**
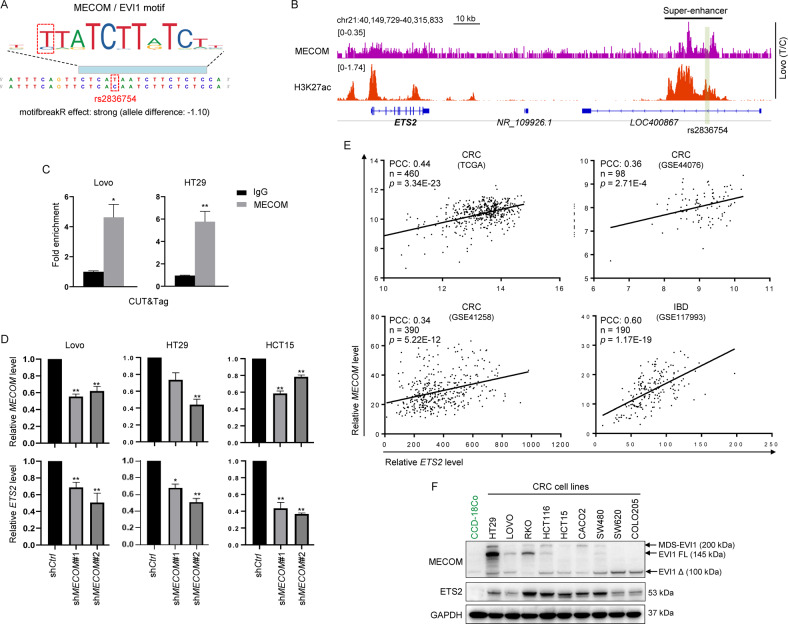
Oncogenic transcription factor MECOM is recruited to the *ETS2*-SE for *ETS2* transcription activation. **A** Result of motifbreak analysis of the IBD-risk SNP (rs2836754) in *ETS2*-SE showing preferential binding of MECOM/EVI1 at T allele over C allele. **B** IGV tracks showing the MECOM and H3K27ac ChIP-seq profiles across indicated region in Lovo cells. **C** Enrichment of MECOM binding at the IBD-risk SNP (rs2836754) site in *ETS2*-SE as determined by CUT&Tag followed by qPCR analysis. *p < 0.05; **p < 0.01. **D** Expression of *MECOM* and *ETS2* in CRC cells stably expressing shRNAs targeting *MECOM* or a non-targeting shRNA were examined by RT-qPCR. *p < 0.05; **p < 0.01. **E** Scatter plots showing the correlation between *MECOM* and *ETS2* levels in CRC and IBD samples. PCC Pearson Correlation Coefficient. **F** Western blot results showing the expression of MECOM and ETS2 in CRC cell lines and a normal colonic epithelial cell line CCD-18Co. GAPDH was used as a loading control. Bands representing different isoforms of MECOM/EVI1 protein were indicated by black arrows.

To study the mechanism of the eQTL in distal SE in modulating *ETS2* transcription, we first evaluated the effect of the variant on transcription factor binding sites by a well-developed algorithm motifbreakR [29], which interrogates the functional impact of polymorphism within genome intervals with established transcription factor binding motifs. As a result, we found that the predicted affinity of a well-known oncogenic transcription factor MECOM (MDS1 and EVI1 complex locus) to the site was different between genotypes, with a stronger preference to the reference allele (T) over the alternative allele (C) (Fig. 4A). Given that *ETS2* expression was significantly higher in CRC patients with T/T genotype than the ones with C/C genotype at the eQTL (Fig. 3F), it was reasonable to hypothesize that MECOM activates *ETS2* transcription through binding to its distal SE. Indeed, ChIP-seq experiments revealed the enrichment of MECOM binding across the *ETS2*-SE (Fig. 4B) and CUT&Tag (Cleavage Under Targets and Tagmentation) experiments confirmed its recruitment to the eQTL site (Fig. 4C) in two CRC cell lines with heterozygous alleles (Supplementary Fig. 4A). Importantly, knock-down of *MECOM* in three different CRC cell lines resulted in significant downregulation of *ETS2* expression, suggesting that MECOM is required for *ETS2* activation in CRC. In concert with these findings, the level of *ETS2* was found to be strongly correlated with the level of *MECOM* in both CRC and IBD samples from large cohort studies (Fig. 4E). Interestingly, the protein level of ETS2 was correlated with the level of full length EVI1 and MDS-EVI1 fusion protein rather than a shorter isoform of EVI1 (EVI1 Δ) (Fig. 4F), indicating that the full function EVI1 protein especially the N-terminal zinc finger domains is necessary for *ETS2* activation.

### MECOM and ETS2 are required for sustaining the oncogenic capacity of CRC cells

**Fig. 5:**
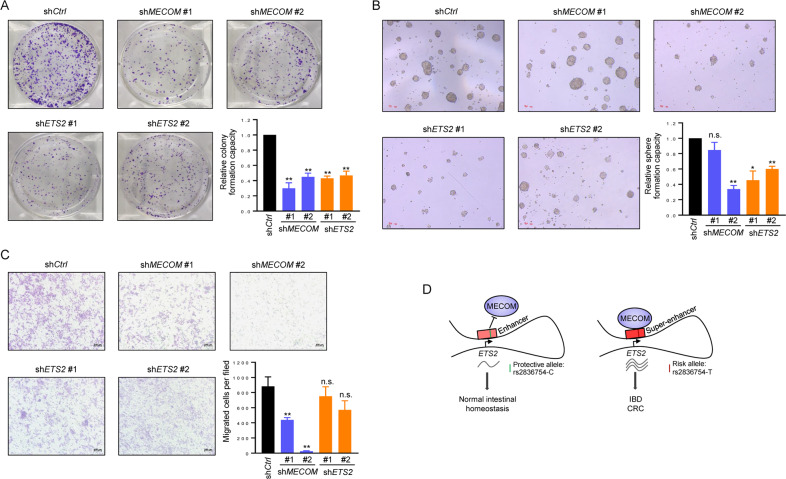
MECOM and ETS2 are required for sustaining the oncogenic capacity of CRC cells. **A** Representative photos and quantification results of monolayer colony-formation assay for HCT15 cells stably expressing shRNAs targeting *MECOM*, *ETS2* or a non-targeting shRNA. **B** Representative photos and quantification results of sphere-formation assay for HT29 cells stably expressing shRNAs targeting *MECOM*, *ETS2* or a non-targeting shRNA. **C** Representative photos and quantification results of transwell migration assay for Lovo cells stably expressing shRNAs targeting *MECOM*, *ETS2* or a non-targeting shRNA. *p < 0.05; **p < 0.01; n.s. non-significant. **D** Schematic diagram showing the mechanism underlying the activation of oncogenic *ETS2* by a distal SE in IBD and CRC. Please refer to the text for details.

Although MECOM has been widely studied for its oncogenic roles in various cancers [30, 31], its functional contribution to colorectal tumorigenesis is still not fully understood. Meanwhile, the role of ETS2 in CRC remained poorly characterized. Consistent with earlier findings [32], we found that *MECOM* was upregulated in colorectal adenomas and primary CRCs (Supplementary Fig. 4B). Remarkably, silencing of *MECOM* or *ETS2* led to the inhibition of both monolayer colony-formation (Fig. 5A) and sphere-formation capacity (Fig. 5B) of CRC cells, while *MECOM* but not *ETS2* was required for maintaining the migration potential of CRC cells (Fig. 5C).

Taken together, our study identified a novel disease-specific SE that regulates *ETS2* transcription via long-range interaction in CRC and IBD and elucidated the mechanism by which an IBD-risk genetic variant affects *ETS2* expression through modulating the recruitment of oncogenic transcription factor MECOM to the *ETS2*-SE (Fig. 5D).

## Discussion

Deciphering the targets and pathways altered by disease-associated variants and cis-regulatory elements (CREs) in the non-coding genome was long been hampered by the lack of precise higher-order chromatin organization information. Fortunately, generation of high-resolution 3D genome maps and comprehensive characterization of promoter-centered chromatin interactions due to recent technology advancements has greatly removed the barriers for us to understand the molecular events outside the coding areas. Our study here established a novel mechanistic link between a distal CRE harboring an IBD-risk variant and oncogenic *ETS2* transcription alteration in CRC and IBD. To our knowledge, this is the first study that decodes, at least in part, the mechanism that accounts for the functional significance of an established IBD risk locus. In fact, enrichment of trait-associated SNPs in enhancers rather than promoters was observed in both IBD [19] and CRC [14]. Therefore, it will be promising to investigate the mechanism of these genetic variations in conferring disease susceptibility under the setting of distal transcription regulation.

Persistent chronic inflammation is a strong risk factor for CRC [33]. Thus, it is of significance to study the molecular alterations occurred in both IBD and CRC, which will shed light on the mechanisms underlying the early development and pathogenic evolution of CRC. This work pinpointed ETS2 as a potential moderator for an IBD-risk SNP in generating predisposition to IBD and CRC. Intriguingly, the ETS2 binding sites was found to be overrepresented in the promoter regions of genes upregulated both in experimental colitis and IBD patients [34]. Moreover, ETS2 was reported to play vital roles in maintaining persistent inflammatory response in macrophages and neutrophils of mice [35, 36] and inducing proinflammatory phenotype in endothelial cells [37]. In concert with these findings, our study showed that *ETS2* was upregulated in IBD and higher *ETS2* expression predicted resistance to infliximab (α-TNFα) treatment for IBD (Supplementary Fig. 4C, D). ETS2 belongs to a large and evolutionarily conserved ETS transcription factor family, which mainly exerts its function by directly regulating the transcription of downstream genes [21, 38]. Therefore, we can speculate that the activation of ETS2 by distal SE promotes the transcription of its downstream genes that drive inflammatory response, thereby conferring susceptibility to IBD and CRC development. Further studies using acute/chronic colitis and colitis-associated cancer models with genetic perturbation of *ETS2* will clarify its contribution to inflammation-driven cancer.

## Materials and methods

### Chromosome conformation capture (3C) assay

3C assays were performed as previously described [39]. In brief, cells were fixed with 2% formaldehyde for 5 min at room temperature and quenched by 2 M glycine. After fixation, cells were incubated with lysis buffer (10 mM Tris-HCl pH 8.0,10 mM NaCl, 0.2% NP-40) at 4 °C for 90 min. The isolated nuclei were then digested with the EcoRI restriction enzyme (Tarkara) at 37 °C for 1 h. The digested DNA is extensively diluted to favor intra-molecular ligations (2 μg digested DNA in 800 μl ligation system) before the fragments were ligated by T4 DNA ligase (Sangon) at 22 °C for 4 h. The resulting chromatin was incubated at 65 °C overnight to reverse the cross-links. The DNA was purified by QIAquick PCR purification Kit (Qiagen) for subsequent PCR analysis. Primers used for 3C-PCR were listed in Supplementary Table 1.

### Super-enhancer and eRNA analysis

For super-enhancer analysis, the ROSE (Rank Ordering of Super-Enhancers) algorism [40] with parameters -s 12500 -t 2000, in which enhancers within 12.5 kb are stitched together, were used. Enhancers above the inflection point of the ranking curve were defined as SEs. Integrative Genomics Viewer (IGV) was used to visualize peaks across the genome. For quantification of ChIP-seq signal intensity at indicated genomic intervals, read intensity normalized by sequencing depth (RPGC) at a bin size of 10 bp was calculated.

### Plasmids and cell lines

shRNAs targeting *MECOM or ETS2* were cloned into pLKO.1-puro vector (Addgene, # 8453). Target sequences of all shRNA plasmids were listed in Supplementary Table 2. The inserts in all plasmids were confirmed by Sanger sequencing.

HT29, Lovo and HCT15 cell lines were obtained from the cell bank of Chinese Academy of Sciences and maintained in RPMI1640 medium supplemented with 10% fetal bovine serum in a 5% CO^2^ incubator at 37 °C. Cell lines used in this study were authenticated by STR profiling and routinely tested for mycoplasma contamination.

### sgRNA directed dCas9-KRAB transcription repression

The sgRNA directed dCas9-KRAB transcription repression system used in this study was used as reported previously [17, 24]. The pLV-hU6-sgRNA-hUbC-dCas9-KRAB-T2a-Puro vector backbone was obtained from Addgene (# 71236). Three sgRNAs targeting different *ETS2-*SE regions were cloned into this vector. Target sequences of sgRNAs were listed in Supplementary Table 2. Lentiviral particles containing the constructs were used to infect CRC cells. The infected cells were then subjected to puromycin selection (2 μg/ml) and collected for expression analysis.

### CUT&Tag

The cleavage under targets and tagmentation (CUT&Tag) was performed according to the manufacturer’s instructions (Novoprotein, #N259-YH0). Briefly, around 10^5^ cells were incubated with 10 μl activated ConA beads at room temperature for 10 min, followed by incaution with 1 μg primary antibody (EVI1: Abcam, ab124934; Normal Rabbit IgG: Cell Signaling Technology, #2729 S) on a rotating platform at room temperature for 2 h, and then with secondary antibody (Abcam, ab182016) at room temperature for 1 hour. The resulting samples were incubated with ChiTag transposome on a rotating platform at room temperature or 1 h and then with Tagmentation buffer at 37 °C for 1 h. After incubation, fragmentation was terminated and the DNA was extracted by phenol-chloroform and ethanol precipitation, which was subsequently analyzed by quantitative PCR for enrichment of MECOM/EVI1 binding at indicated site. Primers used for qPCR were listed in Supplementary Table 1.

### Lentiviral particle production and stable cell line generation

Lentiviral particles were produced using a 3rd generation packaging system with pCMV-VSV-G (Addgene #8454) and psPAX2 (Addgene #12260) in HEK293T cells. Medium was collected 72–96 h after transfection and viruses were concentrated using Lenti-X Concentrator reagent (Clontech). Cells were seeded in 6-well plate one day before infection, and were infected by the lentiviral particles in the presence of 4 μg/ml polybrene. 2 μg/ml puromycin was applied 72 h post-infection for stable cell line selection.

### Quantitative reverse transcription-PCR (qRT-PCR)

Total RNA was extracted by TRIzol reagent (Life Technologies) according to manufacturer’s instructions. One microgram of RNA was used for each reverse transcription reaction, which was set by PrimeScript RT kit with genomic DNA eraser (Takara). qRT-PCR was carried out using SYBR Green PCR Master Mix (Takara) on a qTOWER platform (Jena). Primers used in this study were listed in Supplementary Table 1.

### Western blot

Western blot was carried out as described previously [41]. Briefly, Membranes were incubated with a primary antibody at 4 °C overnight, followed by incubation with a secondary antibody at room temperature for 1 h. The primary antibodies used were anti-ETS2 (Proteintech, 12280-1-AP), anti-EVI1 (Abcam, ab124934), and anti-GAPDH (Proteintech, 10494-1-AP). Images of original full-length western blots were provided in Supplementary Fig. 4.

### Monolayer colony formation, sphere formation and transwell assays

For colony formation assay, cells were seeded in 6-well plate and allowed for growth for two weeks. Medium with puromycin (2 μg/ml) were refreshed every two days. At the end of experiment, colonies were stained by crystal violet. The sphere-formation assay was conducted as described previously [42]. Briefly, dispersed single cells were cultured in serum-free Dulbecco’s modified Eagle’s medium DMEM/F-12 supplemented with 1X B27, 5 μg/mL insulin, 20 ng/ml fibroblast growth factor (FGF), and 20 ng/ml epidermal growth factor (EGF) in ultra-low attachment cell culture plates for five to seven days. The tumor spheres were photographed under a light microscope, and the number of spheres were counted in at least five independent fields for each well. For migration assay, cells in serum-free medium were seed in the upper chamber of transwell insert (Corning) in 24-well plate (1 × 10^5^ cells per well), with medium containing 10% fetal bovine serum in the bottom chamber. After thirty hours of incubation, migrated cells at the bottom side of the insert membrane were stained with crystal violet. At least five random fields were photographed and counted using a phase-contrast inverted microscope.

### Statistical analysis

Data were presented as mean ± standard error of the mean (SEM). All experiments were performed by investigators not blind to group allocation. Unless specified elsewhere, all experiments in this study were conducted in triplicate independently. Difference between two independent groups was analysed by unpaired *t*-test under two-tail hypothesis with equal variance assumption. Differential expression between matched samples was evaluated by paired *t*-test. Difference of multiple groups was evaluated by one-way analysis of variance (ANOVA) and Bonferroni post hoc tests were used to further evaluate results with significance from ANOVA. Statistical analysis was performed in GraphPad Prism 9 or using SPSS 21.0 package.

## Supplementary information

Supplementary materials

Full length western blots

Reproducibility checklist

## Data Availability

All data supporting the findings of this study are presented in the paper. Next-generation sequencing data used in this study were all publicly available with their source and identification numbers indicated in the corresponding figures. Further information is available from the corresponding author upon request.
