# Supplementary material for: A distal super-enhancer activates oncogenic ETS2 via recruiting MECOM in inflammatory bowel disease and colorectal cancer
Source: Cell Death Dis. 2023 Jan 6;14(1):8. doi: 10.1038/s41419-022-05513-1 (PMC9822945; doi:10.1038/s41419-022-05513-1)
Supplement: Supplementary file 1 — Supplementary materials [file 41419_2022_5513_MOESM1_ESM.pdf]

Supplementary Figure legends

Supplementary Figure 1. Plots showing the level of ETS2 mRNA expression in primary CRC samples from TCGA cohort with different clinical and molecular features. MSS, microsatellite stable; MSI-L, microsatellite instability low; MSI-H, microsatellite instability high; CIMP, CpG island methylator phenotype; MMR, mismatch repair.

Supplementary Figure 2. Heatmap showing the expression of *ETS2* in CRC samples (TCGA cohort) together with indicated clinical or molecular attributes. The heatmap was generated by UCSC Xena browser (https://xena.ucsc.edu/).

Supplementary Figure 3. (A) Left panel: PCR results for 3C samples or control samples using primers targeting indicated regions (fragments in *ETS2* promoter or its distal SE). Right panel: Sanger sequencing results of indicated PCR products from 3C experiments. (B) Scatter plots showing the correlation between *ETS2* expression level and H3K4me3 level at *ETS2*-SE or *ETS2* promoter in primary CRC. PCC, Pearson Correlation Coefficient. (C) Scatter plots showing the correlation between *ETS2* expression level and eRNA level at the *ETS2*-SE in primary CRC. PCC, Pearson Correlation Coefficient.

Supplementary Figure 4. (A) Results of Sanger sequencing showing the genotype of the IBD-risk SNP (rs2836754) in indicated CRC cell lines. A peak with mixed base signal represents a heterozygous allele. (B) Relative *MECOM* mRNA expression levels in begin adenomas and primary CRCs with adjacent normal colon as controls. (C) Boxplots showing the expression level

of *ETS2* in IBD samples with differential sensitivity to infliximab (α-TNFα) treatment. (D) ROC curve showing the power of *ETS2* expression level in distinguishing infliximab-resistant from infliximab-sensitive IBD samples. AUC, area under curve.

Supplementary Figure 1

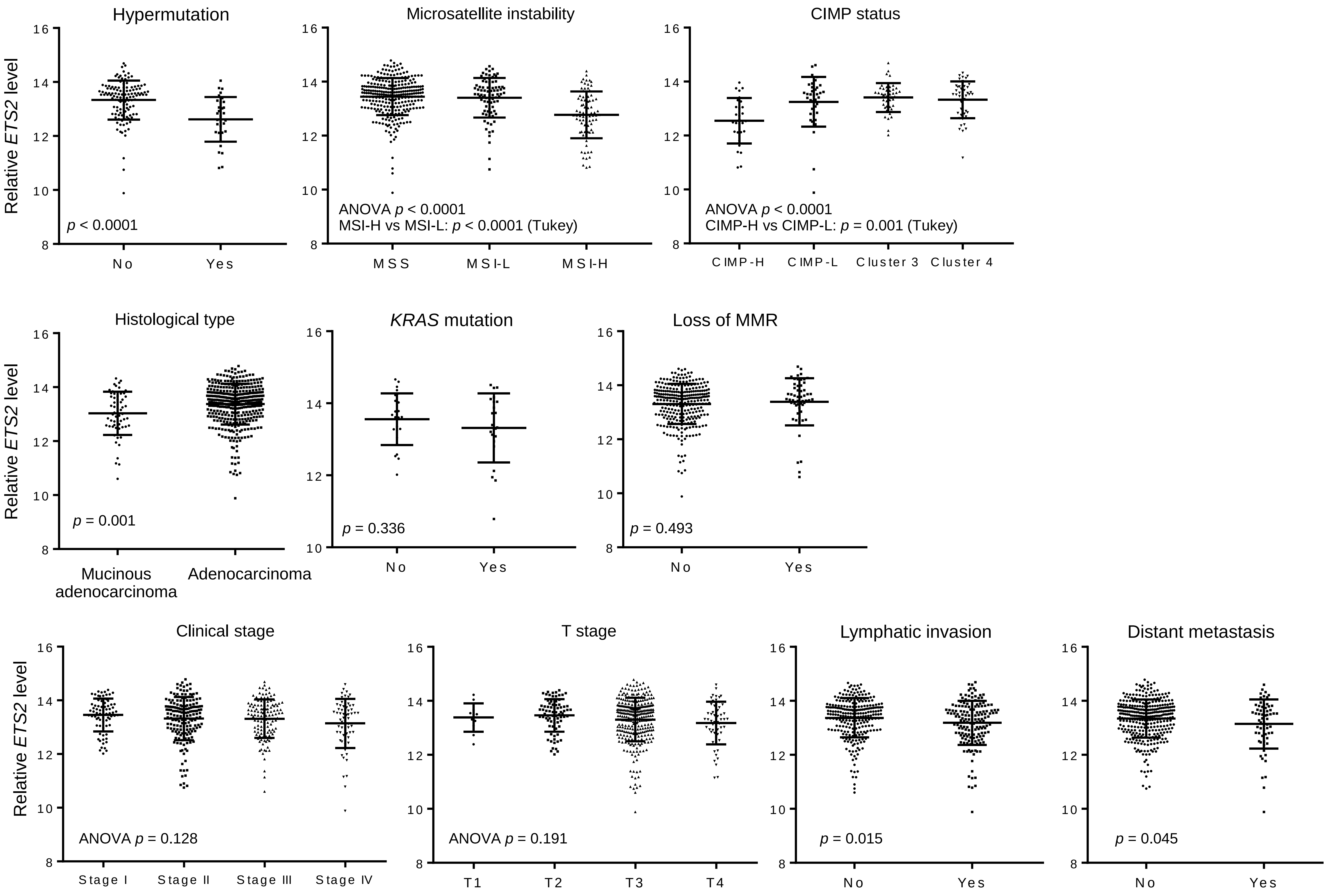

Supplementary Figure 2

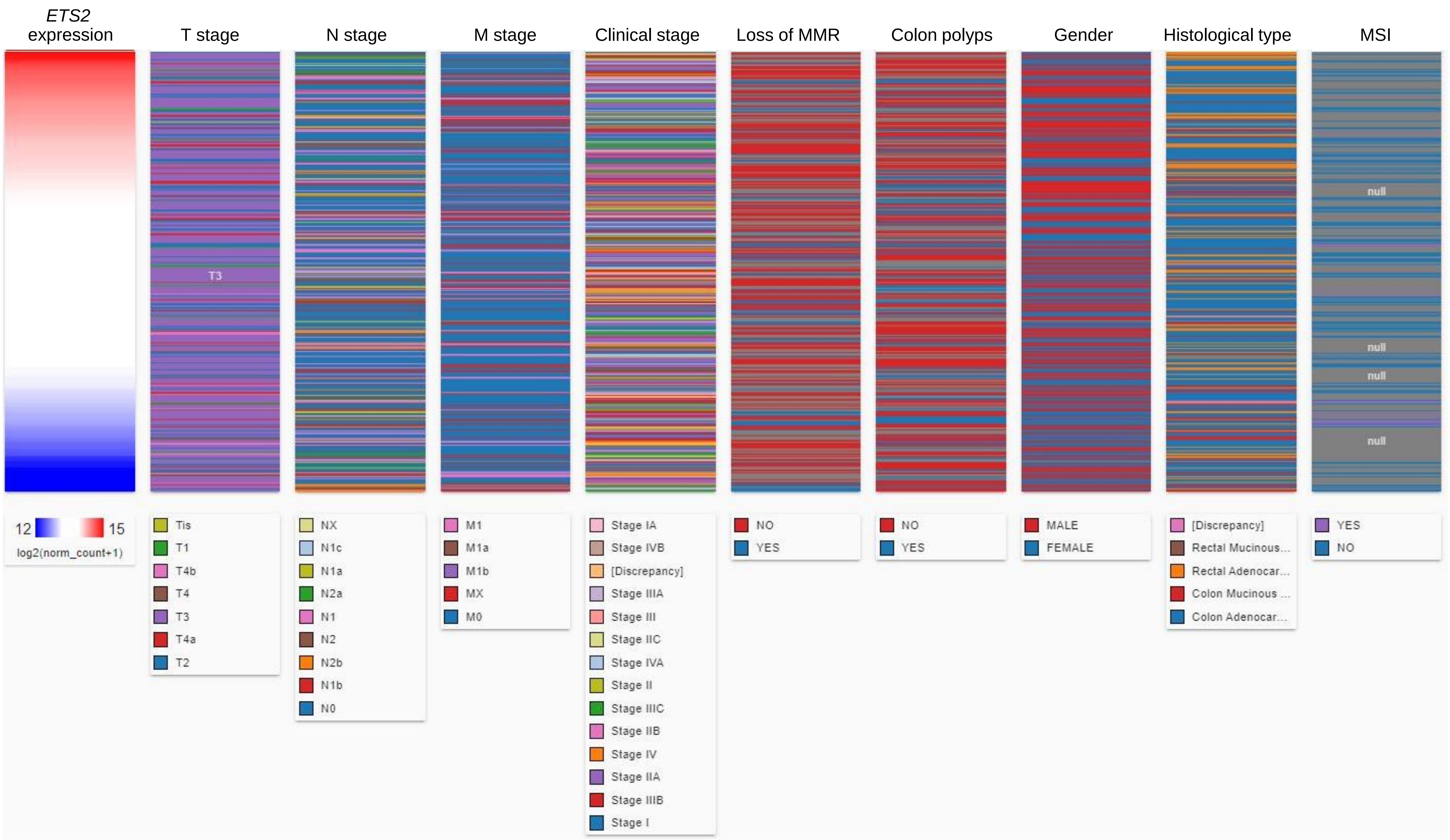

Supplementary Figure 3

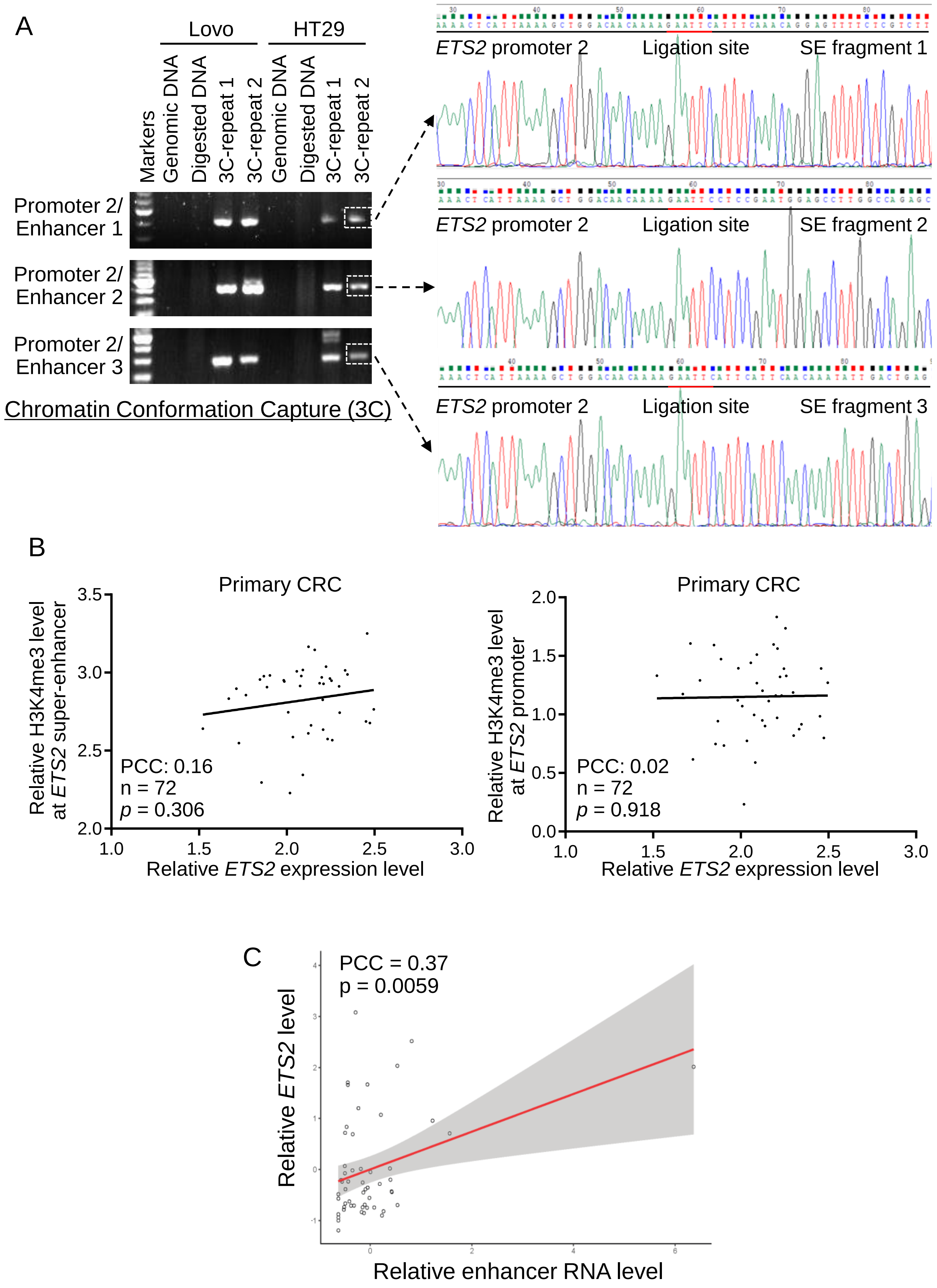

Supplementary Figure 4

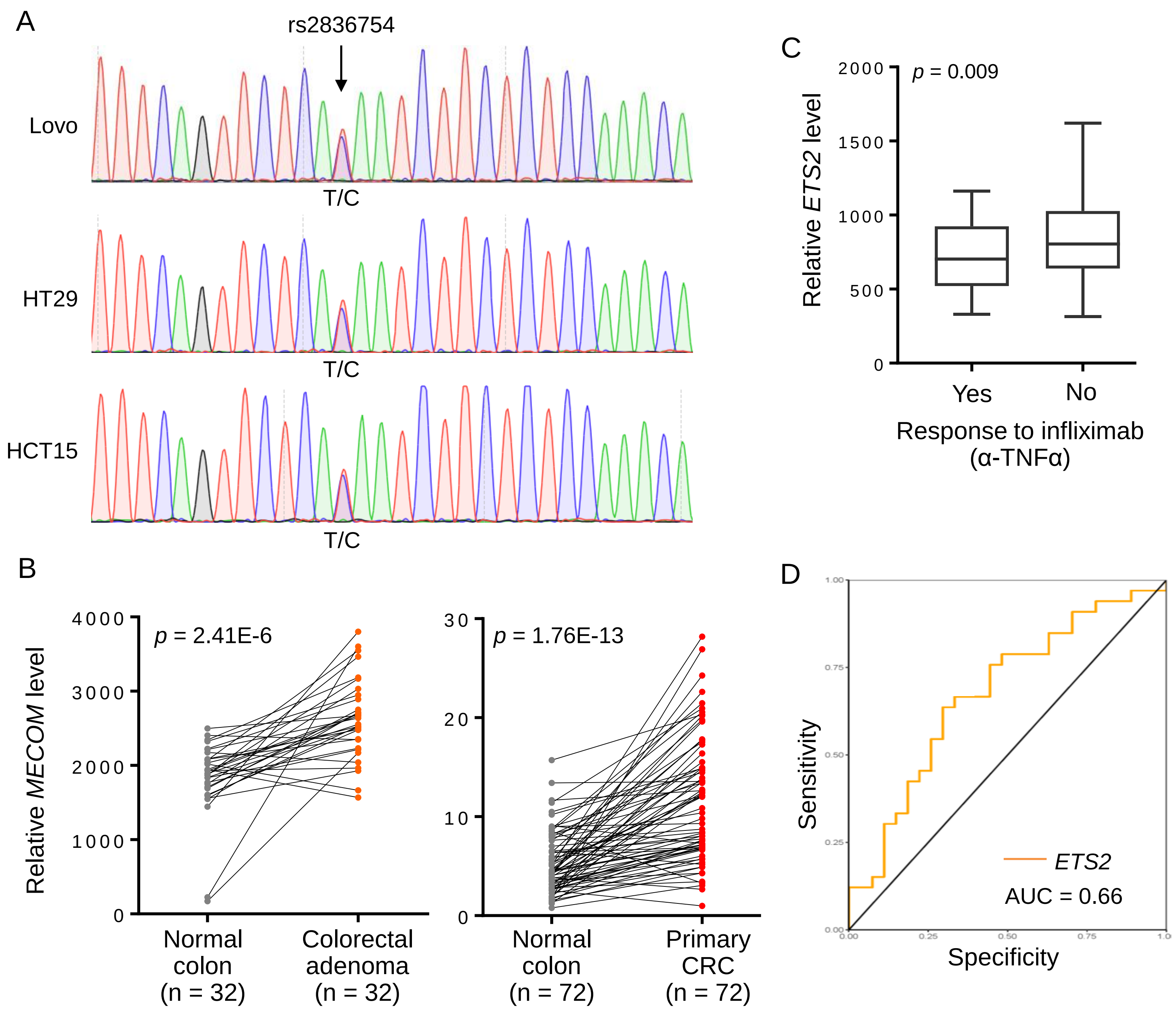

Supplementary Table 1. Primers used in this study.

Primers for RT-qPCR		
**Primer name**	**Sequence (5' to 3')**	**Product size (bp)**
GAPDH-F	GGAGCGAGATCCCTCCAAAAT	196
GAPDH-R	GGCTGTTGTCATACTTCTCATGG	
MECOM-F	TATCCACGAAGAACGGCAATATC	89
MECOM-R	CATGGAAACTTTTGGTGATCTGC	
ETS2-F	CTGGGCATTCCAAAGAACCC	84
ETS2-R	CCAGACTGAACTCATTGGTGG	
**Primers for 3C-PCR**		
**Primer name**	**Sequence (5' to 3')**	**Product size (bp)**
ETS2-promoter 1	AGTTGTGTGTCTGCCTGAAACCATT	214
ETS2-enhancer 1	CCAGGGCTGCTATTTTGAAGGG	
ETS2-promoter 1	AGTTGTGTGTCTGCCTGAAACCATT	393
ETS2-enhancer 2	GGGTTGCTTTTCCACCTAAAGGG	
ETS2-promoter 1	AGTTGTGTGTCTGCCTGAAACCATT	270
ETS2-enhancer 3	TGTTGCACCTGTTTGTGAAGACC	
ETS2-promoter 2	TCAGAGGTTCAAGAATGGGGTCG	225
ETS2-enhancer 1	CCAGGGCTGCTATTTTGAAGGG	
ETS2-promoter 2	TCAGAGGTTCAAGAATGGGGTCG	404
ETS2-enhancer 2	GGGTTGCTTTTCCACCTAAAGGG	
ETS2-promoter 2	TCAGAGGTTCAAGAATGGGGTCG	281
ETS2-enhancer 3	TGTTGCACCTGTTTGTGAAGACC	
Positive control-F	GCCCTTTTTGCCATGTGAGG	611
Postive control-R	CCAGTGCAGCTTGGGTCTTA	
**Primers for CUT&Tag-qPCR**		
**Primer name**	**Sequence (5' to 3')**	**Product size (bp)**
ETS2 SE-SNP-F	TAAGCCACCACTGAAGGTGC	315
ETS2 SE-SNP-R	CAGGGTTCCCGCTTTCTGTA	

Supplementary Table 2. Target sequences of shRNAs and sgRNAs used in this study.

**Name**	**Sequence in sense (5' to 3')**
sh*MECOM* #1	GCGGCAGGATGAATGATTT
shMECOM #2	GGGAACATCTGGAGCAAAT
sh*ETS2* #1	GCACTACGTCTTCCTTAAA
shETS2 #2	GCCTCATTCTGAACTGGAA
sh*Ctrl*	GCTACTATGATCTAGGTGA
sgE1	GGGAGTTGACTTCCACCATG
sgE2	GGGAGGCCCAGTTTCCATCG
sgE3	GAACTACTGTTGCATCAAGA
sgNC	CAACAAACAGTGTTACAGTG
