# Supplementary figures and images for: A distal super-enhancer activates oncogenic ETS2 via recruiting MECOM in inflammatory bowel disease and colorectal cancer
Source: Cell Death Dis. 2023 Jan 6;14(1):8. doi: 10.1038/s41419-022-05513-1 (PMC9822945; doi:10.1038/s41419-022-05513-1)

Supplementary Figure 4

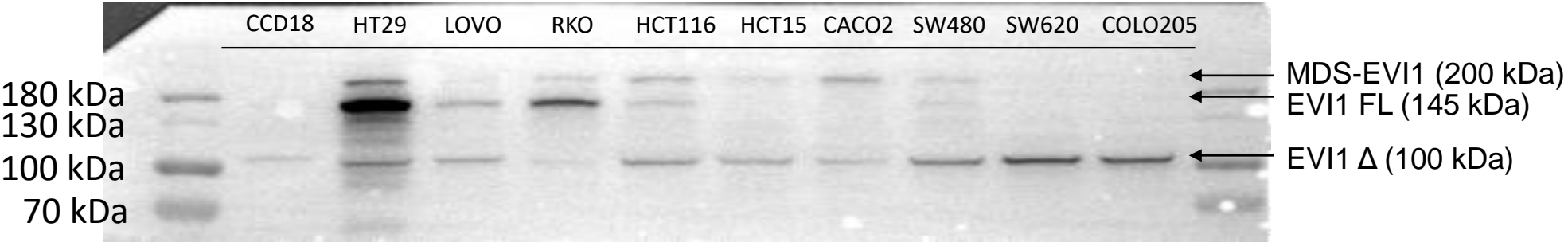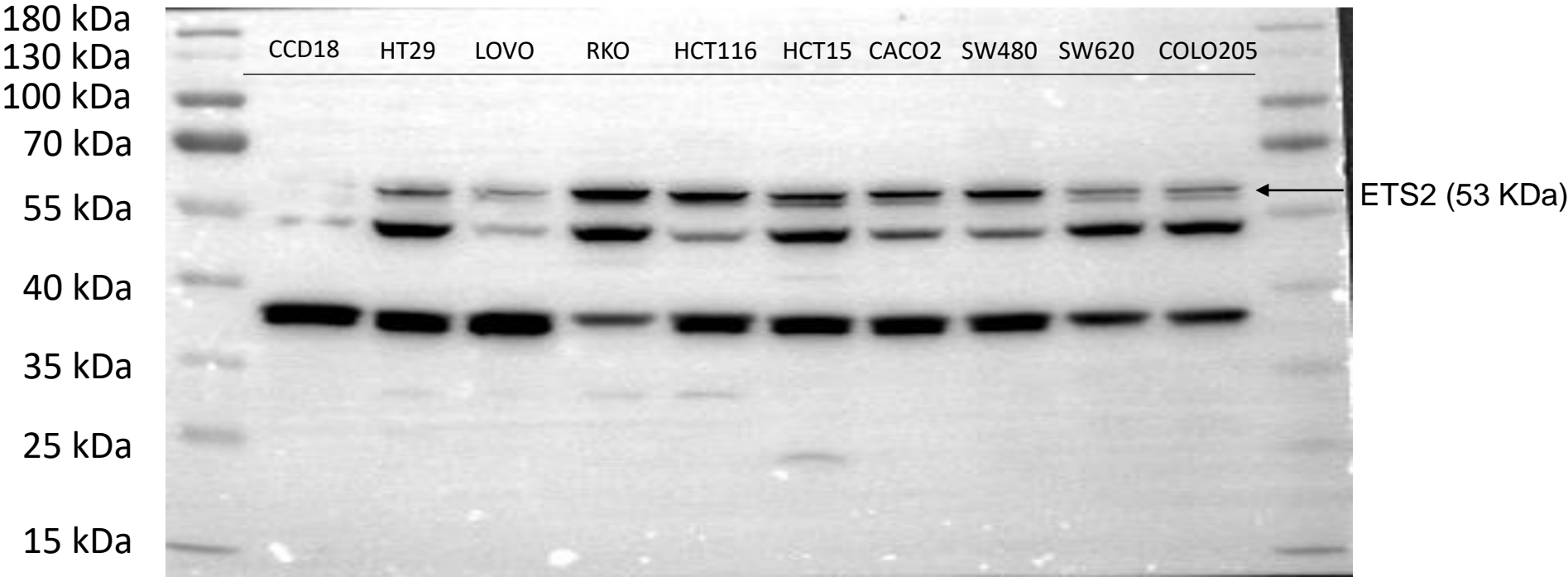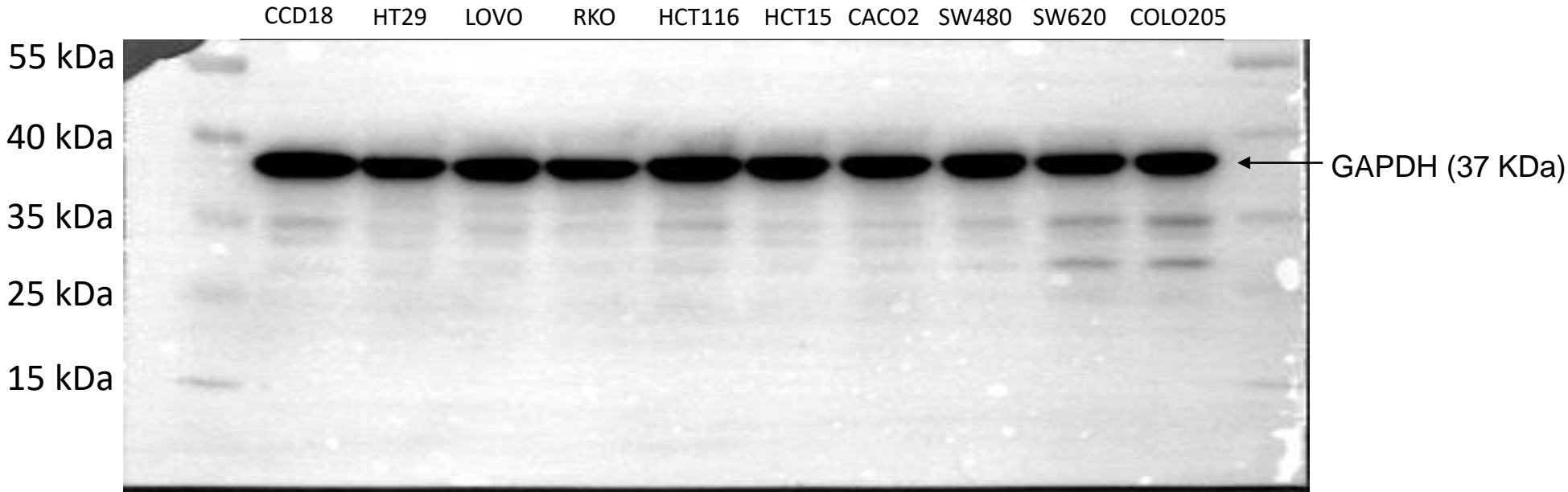

Supplement: Supplementary file 2 — Full length western blots [file 41419_2022_5513_MOESM2_ESM.pdf]
